# Long-term complete response with third-line PARP inhibitor after immunotherapy in a patient with triple-negative breast cancer: a case report

**DOI:** 10.3389/fonc.2023.1214660

**Published:** 2023-08-04

**Authors:** Roberta Caputo, Martina Pagliuca, Matilde Pensabene, Sara Parola, Michelino De Laurentiis

**Affiliations:** ^1^ Division of Breast Oncology, Istituto Nazionale Tumori - IRCCS - Fondazione G. Pascale, Naples, Italy; ^2^ Clinical and Translational Oncology, Scuola Superiore Meridionale (SSM), Naples, Italy; ^3^ U981 Molecular Predictors and New Targets in Oncology, Gustave Roussy, Villejuif, France; ^4^ Unità Operativa Oncologia del PO di San Felice a Cancello, Caserta, Italy

**Keywords:** advanced breast cancer, *BRCA* mutation, triple negative breast cancer, PARP inhibitor, immunotherapy

## Abstract

While standard treatment has shown efficacy in patients with breast cancer gene (*BRCA*) mutations, recurrence rates are high and additional effective therapies are needed. Olaparib, a poly adenosine diphosphate–ribose polymerase (PARP) inhibitor, approved for the treatment of metastatic germline *BRCA1*/*BRCA2* breast cancer (BC), has demonstrated evidence of a progression-free survival (PFS) benefit, good safety profile, and improved quality of life compared with standard chemotherapy. We here describe the case of a patient with *BRCA1* mutated advanced BC and a long history of response to chemotherapy and immunotherapy who received systemic treatment with olaparib. First diagnosed in March 2011 at the age of 38 years with early-stage BC of the right breast, she underwent quadrantectomy plus ipsilateral axillary lymphadenectomy and adjuvant treatments with chemotherapy regimen containing 5-fluorouracil, epirubicin, and cyclophosphamide (FEC) followed by radiotherapy. Five years later, following a contralateral nodule detection leading to left breast quadrantectomy, she received adjuvant systemic treatment with docetaxel plus cyclophosphamide and radiotherapy. Gene testing showed a germline *BRCA1* deleterious variant, and she underwent bilateral prophylactic mastectomy and oophorectomy. One year later, skin metastasis and bone infiltrations were detected, and she was started on first-line systemic treatment. The patient was enrolled in the IMpassion131 trial (investigating atezolizumab addition to paclitaxel) but unblinding showed that she was randomized in the placebo arm. She received second-line systemic therapy with LAG525 plus carboplatin (CLAG525B2101 trial) resulting in a PFS of 14 months. At disease progression, she was eligible for systemic third-line therapy with olaparib (300 mg twice daily) and had a complete response after 6 months of therapy and a PFS of 40 months at the time of writing. To the best of our knowledge, this is the first report of a complete response following treatment with third-line systemic olaparib in a long-responding patient and relatively good tolerability and quality of life, pre-treated with both chemotherapy and immunotherapy.

## 1 Introduction

Despite advances in treatments and increasingly early detection through screening programs, breast cancer (BC) remains the world’s most prevalent cancer with 2.3 million women diagnosed with BC in 2020 and 685,000 deaths (1). BC affects a staggering 1 in 8 women over their lifetime, with an incidence of 109.2 per 100,000 in women aged under 40 (2). The *BRCA1* and *BRCA2* pathogenic variants, identified over 30 years ago, still constitute the most clinically relevant predisposition genes. Although quoted risks vary according to different evidence, data from prospective cohort studies show that the risk for BC is 72% (at age 80) for *BRCA1* mutation (hereafter used as a synonym for pathogenic variant) carriers, and 69% for *BRCA2* mutation carriers (3). Patients diagnosed with BC associated with *BRCA* pathogenic variants frequently suffer from aggressive, high-risk disease since recurrence rates are high, despite standard-of-care treatments including surgery, radiation, and chemotherapy/immunotherapy. There remains a large unmet need for additional novel targeted therapies that produce improved and long-lasting outcomes in this patient population.

*BRCA1* and *BRCA2* are tumor-suppressor genes that encode proteins involved in the repair of DNA double-strand breaks through the homologous recombination repair (HRR) pathway. BCs associated with *BRCA* mutations are more prone to double-strand DNA breaks that cannot be repaired because of a defective HRR pathway. Poly adenosine diphosphate–ribose polymerase (PARP) enzymes are fundamental to repair DNA single-strand breaks, and cells that lack functional *BRCA1*/*BRCA2* are sensitive to PARP inhibition. PARP inhibitors target cancers with defects in HRR leading to synthetic lethality and cancer cell apoptosis (4). Olaparib, an orally administered PARP inhibitor, was approved for metastatic BC by the United States Food and Drug Administration (FDA) (January 2018) and for locally advanced/metastatic BC by the European Medicines Agency (EMA) (April 2019), based on the positive results of the randomized, controlled, open-label, multicenter, international, phase 3 OlympiAD trial (5, 6). The study enrolled patients with a germline *BRCA* (g*BRCA*) mutation and human epidermal growth factor receptor type 2 (HER2)-negative metastatic BC who received no more than two previous chemotherapy regimens for metastatic disease (7). Results showed that monotherapy with olaparib (205 patients assigned to olaparib arm 300 mg twice daily) provided a significant benefit over standard therapy of the physician’s choice (TPC; capecitabine, eribulin, or vinorelbine in 21-day cycles, 97 patients). Median PFS was 2.8 months longer (7.0 vs. 4.2 months) and the risk of disease progression or death was 42% lower with olaparib monotherapy than with TPC. Importantly, quality of life (QoL) consistently improved with olaparib vs. TPC, with a higher proportion of olaparib-treated patients rating their best overall response as “improvement” (33.7% vs. 13.4%). Most adverse events (AEs) in the intervention arm were grade 1/2, and the proportion of patients reporting grade 3 or higher AEs was lower with olaparib (38.0%) than with TPC (49.5%). Olaparib dose interruptions did not significantly affect treatment duration, and few patients discontinued olaparib therapy because of AEs (<5%). In the present manuscript, we report the case of a patient with *BRCA1* mutated (*BRCA1*m) advanced BC who received systemic treatment with olaparib after a long history of response to chemotherapy and immunotherapy.

This case report follows the CARE Guidelines (8).

## 2 Case report

**Figure 1:**
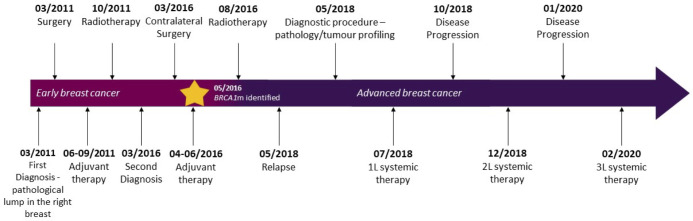
Case report timeline. 1L, first line; 2L, second line; 3L, third line.

**Figure 2:**
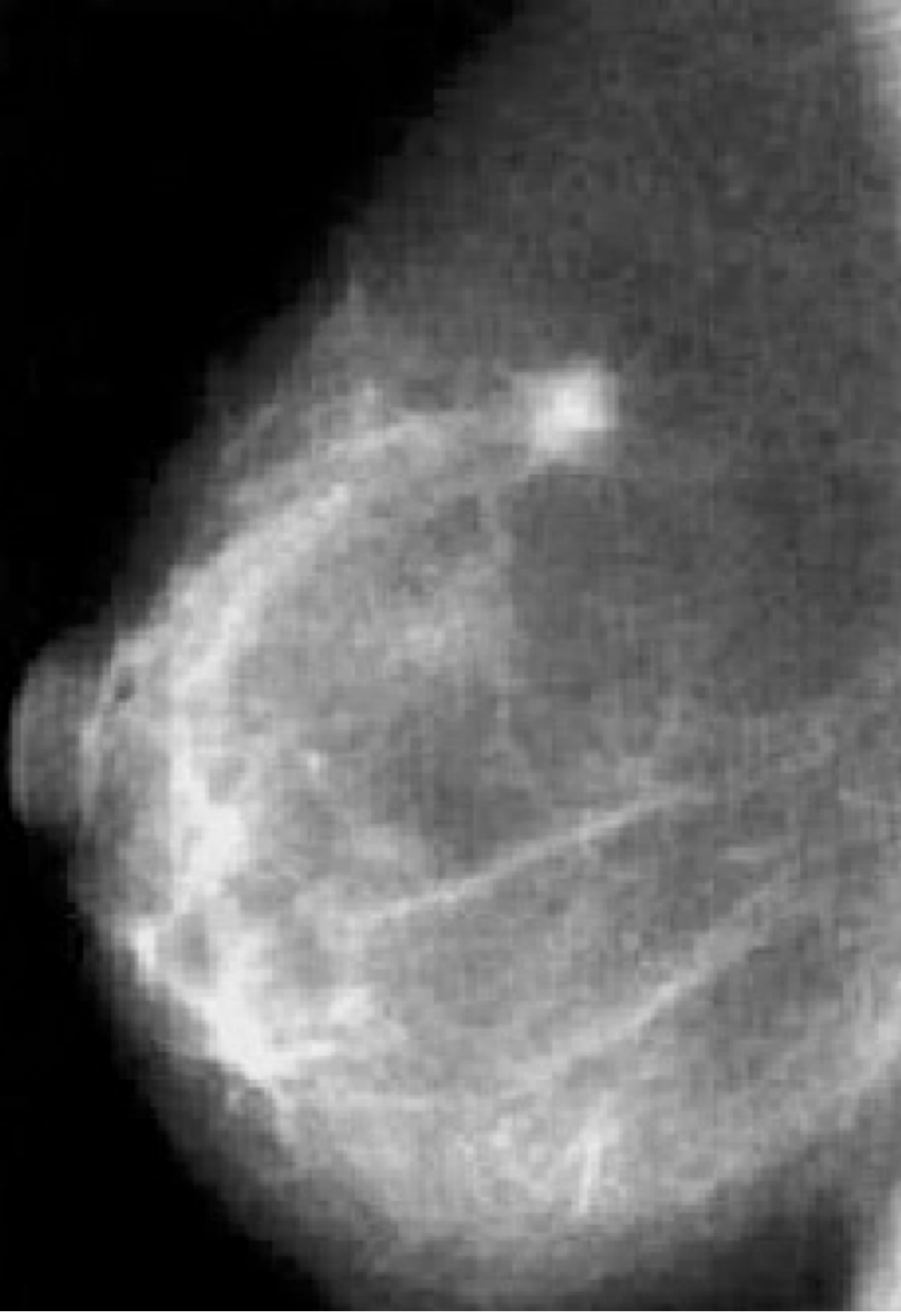
Baseline mammography showing pathological lump in the right breast.

Our patient is a Caucasian woman without relevant personal medical history, except for a mild bronchial asthma seldom treated with low-dose corticosteroids. In March 2011, at the age of 38 years, she was first diagnosed with early-stage BC of the right breast (**Figure 1**). Breast and axillary lymph node ultrasound scan showed a nodule with irregular margins (23 mm diameter) in the upper-outer quadrant of the right breast. Bilateral mammography demonstrated radiopacity in the right breast and fine-needle aspiration biopsy retrieved malignant cells (C5) (**Figure 2**). Subsequently, core needle biopsy showed an infiltrating ductal carcinoma (ER: 0%, PgR: 0%, Ki-67: 30%, HER2-neu: 0), and tumor markers were as follows: CA-15.3 20.7 U/ml, CEA 2.1 ng/ml. No distant metastases were detected. Thus, she underwent right breast-conserving surgery and ipsilateral axillary lymphadenectomy. Postoperative TNM classification was pT2 (28mm) G3 pN1a (1+/20). She received adjuvant treatment (5-fluorouracil 500 mg/m^2^, epirubicin 100 mg/m^2^, and cyclophosphamide 500 mg/m^2^, three cycles, every 21 days, and subsequent docetaxel 100 mg/m^2^, three cycles, every 21 days) and radiotherapy to the right breast.

Follow-up was negative until March 2016 when a second contralateral nodule (15 mm) was identified. Again, there was no evidence of distant metastases. Breast-conserving surgery (left breast quadrantectomy) and ipsilateral axillary sampling were performed [postoperative pT1c 18 mm, pN0 (0/10), ER: 0%, PgR: 0%, Ki-67: 75%, HER2-neu: 0], followed by treatment with docetaxel 600 mg/m^2^ plus cyclophosphamide 75 mg/m^2^ for four cycles every 21 days and subsequent radiotherapy.

**Figure 3:**
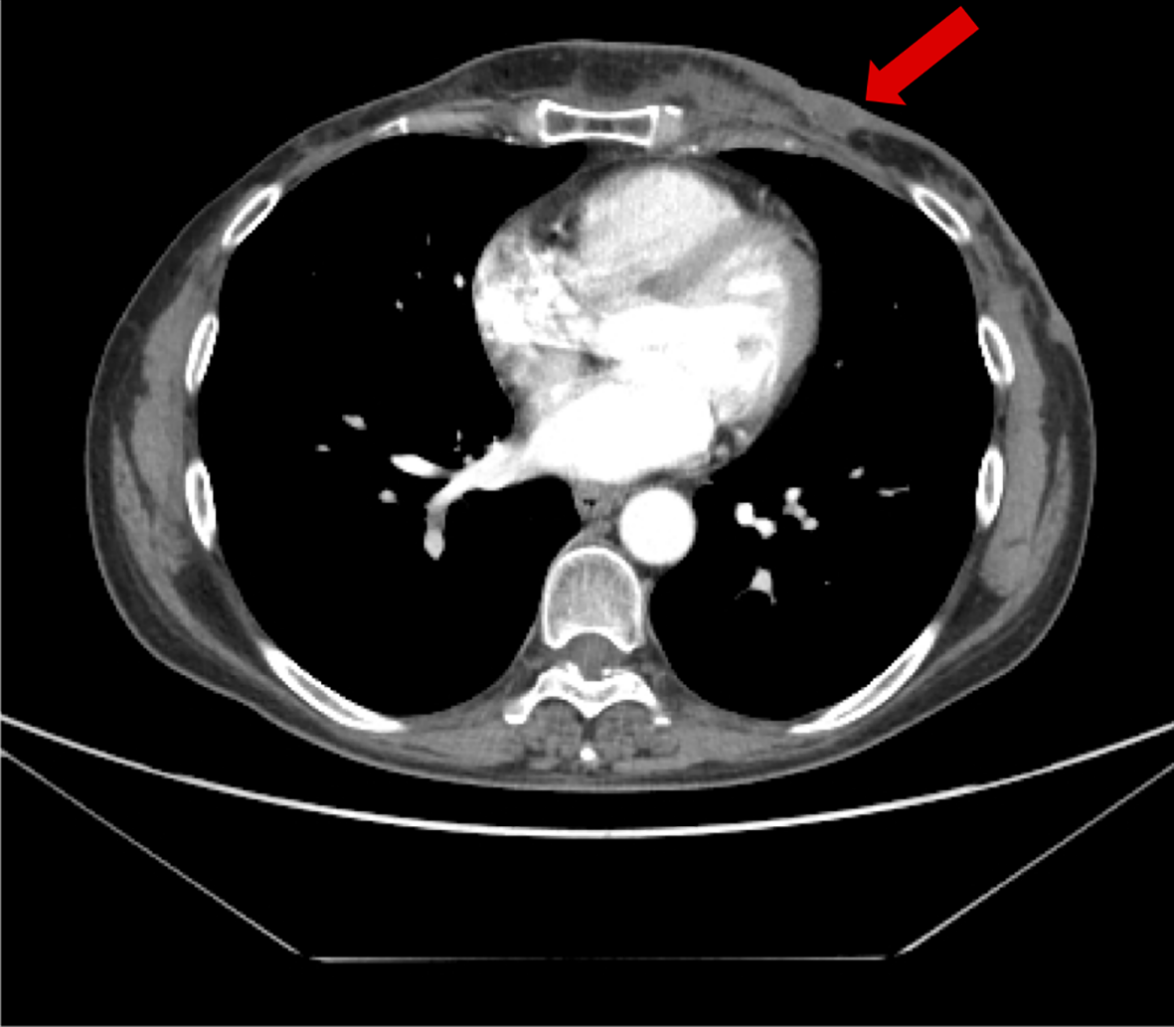
First evidence of metastatic disease on baseline PET-CT scan.

She was referred to the Hereditary Cancer Genetics Clinic at the University Federico II in Naples, Italy, due to the early-onset metachronous BC and the family history including breast and ovarian cancer (grandmother). In May 2016, the result of germline *BRCA* gene testing showed the deleterious variant c.3514G>T p.Glu1172* in the *BRCA1* gene. She underwent bilateral prophylactic mastectomy and oophorectomy in June and July 2017, respectively. She was disease-free for 1 year; then, in May 2018, the PET-CT scan showed involvement of skin of the left breast, increased uptake in multiple lymph nodes of the left axilla and left internal mammary, and bone infiltrations (fourth and sixth left ribs) (**Figure 3**). Pathological examination showed skin metastasis from breast carcinoma (ER: 0%, PgR: 0%, Ki-67: 80%, HER2-neu: 0). Tumor serum markers were within the normal values: CA-15.3 13.9 U/ml, CEA 2.1 ng/ml.

In July 2018, she was enrolled in the phase III IMpassion131 trial investigating treatment with chemotherapy and immunotherapy [atezolizumab 840 mg or placebo by intravenous infusion on Days 1 and 15 ( ± 3 days) every 28 days along with paclitaxel 90 mg/m^2^ on Days 1, 8, and 15 every 28 days until disease progression or unacceptable toxicity]. She received four cycles of therapy. Trial unblinding at disease progression showed that the patient was in the placebo arm.

**Figure 4:**
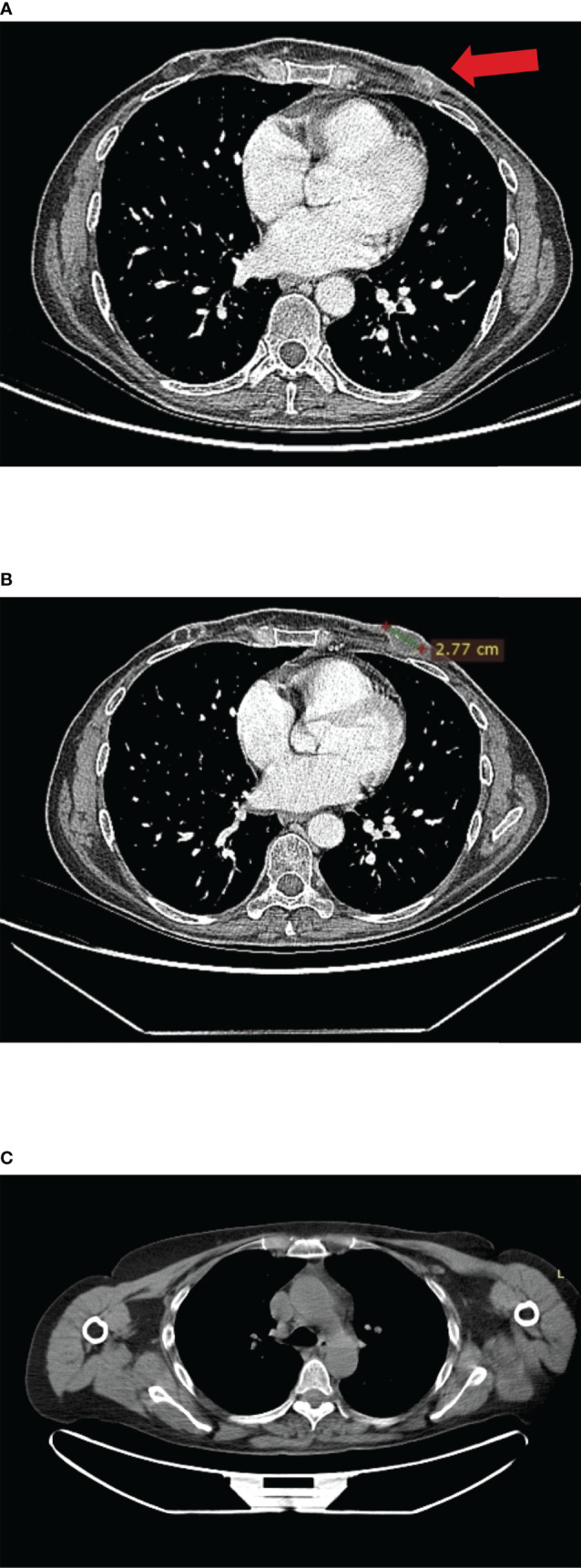
**(A)** CT scan images of left chest wall disease progression at enrolment in the IMpassion131 trial. **(B)** CT scan images at enrolment in CLAG525B2101 trials. **(C)** Complete response after 6 months of treatment with olaparib; no contrast enhancement at PET-CT scan.

In December 2018, she developed mild cough and dyspnea, and chest CT showed the left chest wall disease progression. Thus, she discontinued the ongoing treatment for metastatic BC and was enrolled in the CLAG525B2101 trial investigating LAG525 in combination with spartalizumab, or with spartalizumab and carboplatin, or with carboplatin (**Figure 4A**). LAG525 is a humanized IgG4 monoclonal antibody, acting as a checkpoint inhibitor that binds the Lymphocyte-activation gene 3 (LAG-3) protein and prevents its interaction with class II major histocompatibility complex (MHC-II) molecules. While spartalizumab is a monoclonal antibody directed against the human programmed death-1 (PD-1) receptor, it also inhibits immune checkpoint. The patient received LAG525 (400 mg every 21 days) in combination with carboplatin (AUC 6 every 21 days), both intravenously (iv) for a total of 17 cycles until disease progressed. The PFS was 14 months. AEs included thrombocytopenia grade 3 (G3), diarrhea G1, vomiting G1, and hypothyroidism G2; carboplatin was discontinued after 10 cycles due to infusion-related hypersensitivity reaction.

In February 2020, as a patient with a germline *BRCA* mutation who had received no more than two previous chemotherapy regimens for metastatic disease, she was eligible for olaparib (300 mg twice daily) therapy (**Figure 4B**). Treatment was well tolerated apart from G2 anemia, which imposed a dose reduction—the olaparib dose after 7 months was 100 mg plus 150-mg tablets twice daily. Other reported AEs were nausea, vomiting, and diarrhea (all G1). There was no evidence of increased uptake on PET/CT scan (**Figure 4C**). Treatment with olaparib produced a complete response after 6 months, and at the time of writing, the patient had a PFS of 40 months. It should be, moreover, emphasized how grateful the patient was to be able to receive an effective therapy that did not negatively affect her QoL. Indeed, she had been aware of the advanced stage of disease since she was 45, and that the PFS with the first line of treatment was only 5 months. The complete disease response achieved with a long-lasting and overall well-tolerated oral treatment also increases adherence to the therapy itself, enhancing its efficacy.

## 3 Discussion and conclusion

The diagnostic and therapeutic landscape of BC has changed dramatically in recent years, leading to the introduction of systemic targeted therapies that improve response rates and prolong survival, while maintaining QoL. Our patient was first diagnosed with early BC at the age of 38 years. First-line therapy as part of the IMpassion131 trial was weekly paclitaxel plus placebo (without atezolizumab) and PFS was 5 months. The IMpassion131/130 trials investigated the benefit of adding the immune stimulating agent atezolizumab to a taxane backbone chemotherapy in the first-line treatment of metastatic triple-negative BC (TNBC) (9, 10). Results from the IMpassion130 study indicated improved PFS and clinically meaningful overall survival (OS) benefit with atezolizumab plus nab-paclitaxel in patients with programmed death-ligand 1 (PD-L1)-positive disease and that atezolizumab plus nab-paclitaxel may constitute an important therapeutic option in this disease with high unmet need. However, these results were not replicated in the follow-up IMpassion131 study—atezolizumab plus paclitaxel did not improve PFS or OS in the intention-to-treat (ITT) population or the PD-L1-positive group vs. paclitaxel alone. Second-line therapy was immunotherapy with LAG525, which inhibits LAG-3 (an inhibitory immunoreceptor linked to reduced T-cell proliferation and cytokine production), plus carboplatin (11). Overall, 17 cycles were administered (carboplatin was discontinued after 10 cycles due to AEs) and PFS was 14 months. Olaparib was administered as a third-line therapy based on the results of the landmark OlympiAD trial that showed it to improve PFS and, in addition, to reduce hospitalization (advantage of oral therapy vs. iv) as well as ameliorate QoL, compared with chemotherapy (7, 12). Since then, the OlympiA study demonstrated “practice-changing results”—the first to report the benefits of an adjuvant PARP inhibitor for early stage of germline *BRCA1/2*-mutation-associated BC. This phase III, double-blind, randomized trial enrolled patients with HER2-negative early BC with *BRCA1* or *BRCA2* germline pathogenic or likely pathogenic variants and high-risk clinicopathological factors, who had received local treatment and neoadjuvant/adjuvant chemotherapy. Patients who received olaparib after a median follow-up of 3.5 years experienced a 37% reduction in invasive disease-free survival, including local and metastatic recurrence of BC, other new cancers, and death due to any cause. Among patients with high-risk, HER2-negative early BC with germline *BRCA1/BRCA2* deleterious variants, adjuvant olaparib, after completion of local treatment and neoadjuvant/adjuvant chemotherapy, was associated with significantly longer survival free of invasive or distant disease than placebo. More importantly, a benefit in OS rate was reported with the use of olaparib in this population in an adjuvant setting (4-year OS Δ 3.4%, 95% CI 0.1% to 6.8%). The principal investigator of the study concluded that “patients who received olaparib after surgery and chemotherapy were more likely to be alive without cancer, [as well as] avoid metastasis, than the patients who received placebo—after 10 years of evaluation of PARP inhibitors in BC, a therapy that could likely save many lives is finally at hand” (13, 14). It should be acknowledged that, to date, we have some evidence of the efficacy of PARP inhibitors in the treatment of non-germline *BRCA*m advanced BC. The randomized controlled phase II S1416 trial reported the addition of veliparib to platinum chemotherapy to be effective in metastatic germline *BRCA*-wildtype TNBC with a BRCA-like phenotype, namely, with homologous recombination deficiency (HRD) leading to genomic instability (15). Olaparib monotherapy was further evaluated in the phase II TBCRC 048 trial in a population of patients with advanced BC with germline (other than *BRCA*) or somatic (including *BRCA*) pathogenic variants in DNA damage response pathway genes. Objective responses to the PARP inhibitor were reported in patients with somatic *BRCA1/2* or germline partner and localizer of BRCA2 gene (*PALB2*) mutations (16). In conclusion, our case report shows that in a patient with metastatic BC, third-line therapy with olaparib can lead to a rapid and durable response with clinical complete remission with a relatively good quality of life.

## Data availability statement

The raw data supporting the conclusions of this article will be made available by the authors, without undue reservation.

## Ethics statement

Written informed consent was obtained from the individual for the publication of any potentially identifiable images or data included in this manuscript.

## Author contributions

RC and MPe conceptualized the manuscript. RC, MPa, and SP wrote the original draft of the manuscript. ML contributed to the radiology images and reviewed the manuscript. All authors substantially contributed to the conception, data acquisition, and interpretation of the report. All authors agreed to be accountable for all aspects of the work, ensuring that questions related to the accuracy or integrity of any part of the work are appropriately investigated and resolved. All authors contributed to the article and approved the submitted version.
